# The Biological Significance of Multi-copy Regions and Their Impact on Variant Discovery

**DOI:** 10.1016/j.gpb.2019.05.004

**Published:** 2020-08-19

**Authors:** Jing Sun, Yanfang Zhang, Minhui Wang, Qian Guan, Xiujia Yang, Jin Xia Ou, Mingchen Yan, Chengrui Wang, Yan Zhang, Zhi-Hao Li, Chunhong Lan, Chen Mao, Hong-Wei Zhou, Bingtao Hao, Zhenhai Zhang

**Affiliations:** 1 State Key Laboratory of Organ Failure Research, National Clinical Research Center for Kidney Disease, Division of Nephrology, Nanfang Hospital, Southern Medical University, Guangzhou 510515, China; 2 Department of Bioinformatics, School of Basic Medical Sciences, Southern Medical University, Guangzhou 510515, China; 3 Key Laboratory of Mental Health of the Ministry of Education, Guangdong-Hong Kong-Macao Greater Bay Area Center for Brain Science and Brain-Inspired Intelligence, Southern Medical University, Guangzhou 510515, China; 4 Center for Precision Medicine, Shunde Hospital of Southern Medical University, Foshan 528399, China; 5 Microbiome Medicine Center, Division of Laboratory Medicine, Zhujiang Hospital, Southern Medical University, Guangzhou 510282, China; 6 Division of Epidemiology, School of Public Health, Southern Medical University, Guangzhou 510515, China

**Keywords:** Multi-copy sequence, Multi-copy region, Genetic study, Variant discovery, High-throughput sequencing

## Abstract

Identification of genetic variants via **high-throughput sequencing** (HTS) technologies has been essential for both fundamental and clinical studies. However, to what extent the genome sequence composition affects variant calling remains unclear. In this study, we identified 63,897 **multi-copy sequences** (MCSs) with a minimum length of 300 bp, each of which occurs at least twice in the human genome. The 151,749 genomic loci (**multi-copy regions**, or MCRs) harboring these MCSs account for 1.98% of the genome and are distributed unevenly across chromosomes. MCRs containing the same MCS tend to be located on the same chromosome. Gene Ontology (GO) analyses revealed that 3800 genes whose UTRs or exons overlap with MCRs are enriched for Golgi-related cellular component terms and various enzymatic activities in the GO biological function category. MCRs are also enriched for loci that are sensitive to neocarzinostatin-induced double-strand breaks. Moreover, genetic variants discovered by genome-wide association studies and recorded in dbSNP are significantly underrepresented in MCRs. Using simulated HTS datasets, we show that false **variant discovery** rates are significantly higher in MCRs than in other genomic regions. These results suggest that extra caution must be taken when identifying genetic variants in the MCRs via HTS technologies.

## Introduction

The completion of the Human Genome Project and the advent of high-throughput sequencing (HTS) technologies have facilitated genetic variant discovery and expedited studies aiming to reveal the relationships between genetic variants and disease and health [1]. By re-sequencing genomes of thousands of individuals, scientists from the 1000 Genomes Project revealed millions of benign  single-nucleotide variants (SNVs) [2], [3]. Combining multiple omics techniques, researchers from The Cancer Genome Atlas program (TCGA) described somatic mutations in 33 types of cancers. These results may ultimately lay the foundation for effective cancer prevention, diagnosis, and individualized therapies [4], [5], [6]. In all, genome-wide association studies (GWAS) have identified over 67,259 genetic variants associated with diseases or other traits [7], [8], [9]. Several databases, including dbSNP [10], dbVar [11], and the European Variation Archive, have been established to facilitate genetics studies by archiving and sharing information on sequence variants [12], [13]. Moreover, variant discovery is now widely used in clinical diagnosis for many diseases [14], [15], [16].

These genomic and genetic studies are fundamentally dependent upon accurate identification of genetic variants, which in turn is influenced by many factors such as sample preparation, sequencing error rate, sequencing depth and instruments, bioinformatics analyses, as well as the genome sequence composition [17], [18], [19], [20], [21]. Most of these factors have been investigated in depth [22], [23], [24]. However, the effect of repetitive sequences, particularly those that are identical or highly similar to sequences located elsewhere in the genome [25], [26], on variant calling has received less attention. Repetitive sequences may cause ambiguous alignments of sequencing reads and consequently incorrect identification of genetic variants. Based on their length, repetitive sequences can be classified as either short tandem repeats or interspersed repeats. From a sequence similarity perspective, repetitive sequences can be classified as identical repeats or divergent repeats. Long identical repeats represent a major challenge for variant discovery [27].

HTS technologies typically use a 2 × 150 paired-end sequencing strategy with an insert size of 300–500 bp [28], [29]. To determine whether repetitive sequences measuring several hundred bp in length affect variant calling, we identified 63,897 multi-copy sequences (MCSs) with a minimum length of 300 bp, each of which resides in at least two multi-copy regions (MCRs) in the human genome. These MCRs account for roughly 1.98% of the genome and overlap with 6782 known genes, suggesting that they are biologically important. Of these 6782 MCR-overlapping genes, 3800 contain MCRs within their UTRs or exons. The remaining 2982 MCR-overlapping genes contain MCRs in their introns. Variant discovery using simulated data showed a very high false discovery rate. Our results strongly suggest that extra caution must be taken when identifying variants for genetic studies and clinical diagnoses.

## Results

### MCRs occupy approximately 2% of the human genome

**Figure 1:**
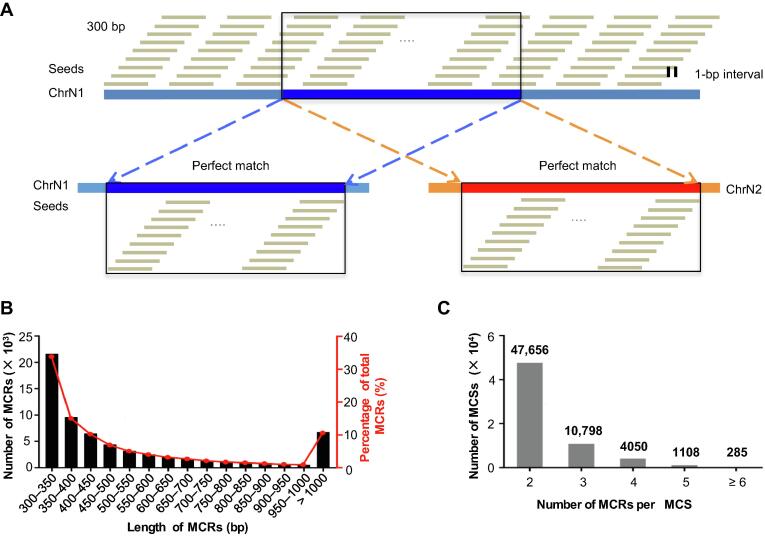
**Identification and chromosomal distribution of theMCRs** **A.** Tiling seed sequences of 300 bp in length with a 1 bp interval (top panel) from ChrN1 are mapped to the reference genome hg19. A set of consecutive seed sequences perfectly mapped to both their origin locus on ChrN1 (blue bar on the bottom left) and another locus on ChrN2 (red bar on the bottom right). These two sequence regions with a length of at least 300 bp are thus defined as MCRs. **B.** Distribution of MCR seeds over different length spans. **C.** Distribution of MCR groups with different members. MCR, multi-copy region.

Firstly, we downloaded the human genome sequence (human genome build hg19) from the UCSC Genome Browser. Secondly, tiling sequences with a length of 300 bp and a 1-bp interval were generated for each chromosome and the mitochondrial genome. We then mapped these tiling sequences back to the same human genome using Burroughs-Wheeler Aligner [30]. Sequences that mapped exactly to multiple loci with no mismatches, insertions, or deletions were extracted as “seeds” for MCSs. If consecutive seeds were perfectly mapped to different loci in succession, they were merged until the continuity was interrupted (Figure 1A). We defined the resultant sequences as MCSs, each of which occupied at least two loci in the genome. Therefore, each set of MCRs shares a single MCS with a length of at least 300 bp.

We identified 63,897 unique MCSs, which mapped to 151,749 MCRs, constituting 1.98% of the human genome (File S1; Tables S1 and S2). A total of 21,609 MCSs (33.82% of the total) are between 300 and 350 bp in length (Figure 1B). The mitochondrial genome contains one MCR of 322 bp (from position 5500 to position 5821), whose counterpart resides on chromosome 1 (from position 566,049 to position 566,370). The longest MCS has a length of 499,419 bp, corresponding to sequences on the X chromosome (from position 2,200,102 to position 2,699,520) and the Y chromosome (from position 2,150,102 to position 2,649,520). Many of the identified MCRs are tandem repeat regions as expected [31]; we observed a high frequency of transposable elements, including LINE/L1 elements (36.98% of the MCRs) and SINEs/Alus (22.56% of the MCRs; Figure S1A). Non-tandem repeat MCRs make up 30,599,033 bp, or approximately 1% of the human genome (Figure S1B).

While 47,656 MCSs correspond to two MCRs, we observed that individual MCSs have as many as 250 copies (Figure 1C). Chromosome 13 possesses the lowest percentage of MCRs (0.34%), and the Y chromosome possesses the highest percentage of MCRs (16.75%). We observed no correlation between the total length of MCRs and the length of the chromosome that harbors them (Figure S2A). In general, more than half of the MCRs consist of sequences from a single chromosome (*i.e.*, they form intra-chromosome pairs) (Figure S2B). More than 80% of the MCSs on chromosomes 5, 9, and 15 form intra-chromosome pairs. However, inter-chromosome pairs are more common for the MCRs on chromosomes 3, 12, 14, and 19. The majority of the MCRs on the X and Y chromosomes are shared between them, and this may be consistent with the hypothesis that the X and Y chromosomes evolved from a pair of identical chromosomes [32]. We also analyzed the relationship between MCRs and known elements in the genome. In all, 38% and 34% of MCRs overlap with pseudogenes and paralogs, respectively, while 13.72% and 8.84% of MCRs overlap with protein-coding genes and lincRNAs, respectively (Table S3). In addition to these types of elements, the MCRs also intersect with various immunoglobulin genes and small RNAs (Table S3).

### MCRs may exert important biological functions

**Figure 2:**
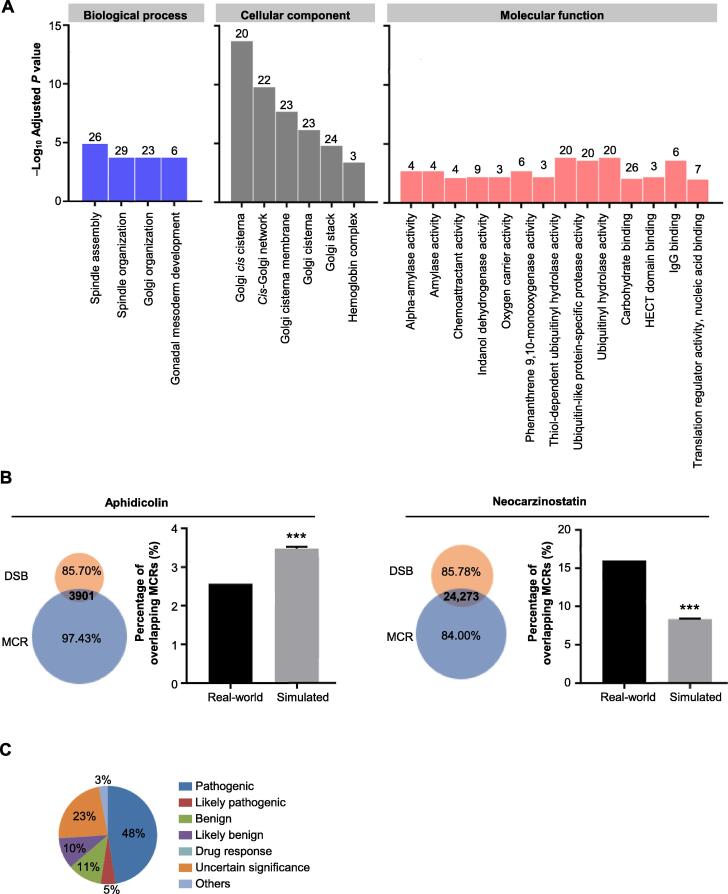
**Biological and clinical significance of MCRs** **A.** GO enrichment analysis of MCR-overlapping genes (adjusted *P* value < 0.01). **B.** Overlap of MCRs with DSBs identified by Crosetto et al. [37] for cells treated with aphidicolin (left) and neocarzinostatin (right). The number in the intersection indicates the number of MCRs overlapping with DSBs. The bar graphs show the enrichment test results for aphidicolin or neocarzinostatin treatment. Real-world dataset contains DSBs overlapping with MCRs, and simulated dataset contains DSBs overlapping with regions randomly chosen from the genome. Data are presented as mean ± SD (*n* = 1000). Chi-Squared test was used for statistical analysis (***, *P* < 0.001). **C.** Clinical significance classification of the ClinVar records in the MCRs. GO, gene ontology; DSB, double-strand break.

Although MCRs constitute approximately two percent of the genome, they overlap with the exons or UTRs of 3800 genes. To see whether these MCR-overlapping genes have specific functions, we performed gene ontology (GO) analysis [33]. Of the 3800 MCR-overlapping genes, 1269 do not have associated GO terms, indicating that many of these genes are not well characterized. The other 2531 genes are enriched for three GO categories (Figure 2A and Figure S3). With a 0.05 cutoff for adjusted *P* values, these genes are enriched in 5, 8, and 25 GO terms in biological process (BP), cellular component (CC), and molecular function (MF), respectively (Figure S3). With a more stringent threshold of *P* ≤ 0.01, the MCR-overlapping genes are enriched for 4, 6, and 13 GO terms in the BP, CC, and MF categories, respectively (Figure 2A).

Among these enriched GO terms, there are six Y-linked testis-specific protein-coding genes, which are expressed in testicular tissue and involved in gonadal mesoderm development (Figure 2A). The MCR-overlapping genes also include six hemoglobin subunits, due to their intrinsic sequence similarity. We also found 23 MCR-overlapping genes that are enriched for Golgi-related terms. Residing at the intersection of the lysosomal, endocytic, and secretory pathways, the Golgi apparatus is an important part of the endomembrane system, which packages proteins into membrane-bound vesicles before sending them to their destination. To accomplish this task, the Golgi membrane contains several classes of enzymes to modify and allocate protein [34]. The MCR-overlapping genes with annotations in the MF category are mainly enzymes, and thus they may be enriched for nucleic acid binding functions or for specific domains through which they can exert enzymatic activities.

To determine whether MCRs play a role in genome stability, we observed the correlation between the MCRs and regions enriched for DNA double-strand breaks (DSBs). Emerging during apoptosis, meiotic, crossing-over, and gene rearrangements, DNA DSBs can be caused by either exogenous or endogenous chemical or physical agents. Unresolved DSBs can lead to genome rearrangements and cause oncogenic mutations such as translocations, deletions, and amplifications [35], [36]. We compared the MCRs with the aphidicolin- and neocarzinostatin-sensitive regions identified by Crosello and colleagues [37]. We observed that 3901 or 24,273 MCRs overlap with DSB regions induced by aphidicolin or neocarzinostatin, respectively (Figure 2B). Enrichment tests showed that the MCRs are significantly overrepresented in the DSBs induced by neocarzinostatin but underrepresented in the DSBs induced by aphidicolin (see Materials and methods and Table S4). This finding indicates that the MCRs may affect genome stability in the context of DSBs.

### Genetic variants in MCRs may cause diseases

After exploring the biological importance of the MCRs, we investigated the clinical significance of known genetic variants located within the MCRs. In this analysis, we focused on the variants in the ClinVar database, which archives the relationships between human variants and phenotypes with supporting evidence. A total of 10,805 genetic variants in the ClinVar database fall within the MCRs. Of these, 5133 (48%) and 525 (5%) of the variants are categorized as pathogenic and likely pathogenic, respectively (Figure 2C). For instance, a single nucleotide mutation in the MCR overlapping *PKD1* causes adult type polycystic kidney disease. In addition, a single nucleotide mutation in the MCR of *TUBG1* leads to complex cortical dysplasia with other brain malformations (*CDCBM*), including aberrant neuronal migration and disrupted axonal guidance. Similarly, GWAS aims to discover single nucleotide polymorphisms (SNPs) that are associated with specific phenotypes. We found 78 of the 67,259 SNPs discovered by GWAS fall within MCRs. These findings suggest that MCRs may play important roles in human health.

Array and target sequencing-based technologies have been widely used to identify genetic variants that impact health and diseases [38], [39]. For example, SureSelect Human All Exon v7 (https://earray.chem.agilent.com) from Agilent serves as a cost-effective hybrid-capture solution, focusing on the interpretable portion of the genome. Four thousand six hundred and forty probes on this array (2.14% of the total) overlap with the MCRs. In addition, 881 probes from the GenetiSure Cancer Array and 626 probes from the Postnatal Research CGH + SNP Array (https://earray.chem.agilent.com) overlap with the MCRs. Thus, our identified MCRs may affect 0.21% and 0.15% of the coverage of genetic aberrations associated with cancer sourced from COSMIC and CGC databases [40] and of intellectual disability and congenital anomalies sourced from the ClinGen and ISCA databases [41].

### MCRs lead to a high false discovery rate in variant calling

Due to the potentially important biological and clinical implications of MCRs, we surveyed the SNVs and mutation frequencies from the 1000 Genomes Project [42], ClinVar [43], and The Cancer Genome Atlas (Tables S5–S7). We observed that the frequency of variants reported in MCRs is lower than that in non-MCRs (Figure S4).

By our initial definition, each MCR is at least 300 bp in length, longer than the typical sequencing read in HTS applications, through which researchers identify and validate genetic variants. To determine whether MCRs affect genetic or genomic variant discovery, we randomly introduced SNVs in the MCRs and their flanking regions and generated simulated HTS datasets with different read lengths and sequencing strategies (see Materials and methods). We then identified SNVs in the simulated datasets as described in the materials and methods section and compared them to the known imputed variants. If an identified SNV was not present in the simulated dataset, then it was deemed as a false positive result. The SNVs that were simulated but not identified were defined as false negative results. Simulated SNVs that were successfully identified were treated as accurate results. The rates of accuracy, false positive and false negative were calculated accordingly.

**Figure 3:**
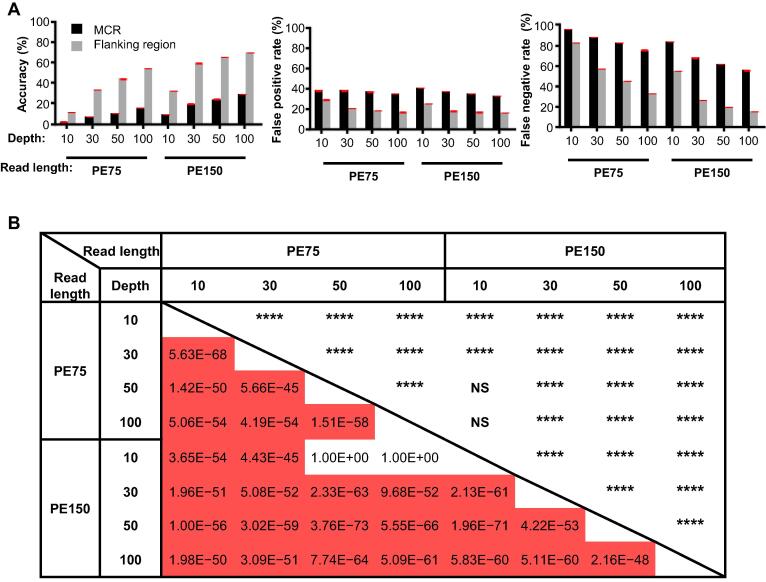
**Variant detection in simulated datasets** **A.** Accuracy and false discovery rates in MCRs and their flanking regions using different sequencing strategies. Data are presented as mean ± SD and error bars are shown in red. Left panel, Accuracy rate; Center panel, False positive rate; Right panel, False negative rate. Mean and standard deviation are shown in Table S8. **B.** Statistical differences in variant detection accuracies among different sequencing strategies. NS, not significant; PE, paired-end sequencing. Independent samples *t*-test was used for statistical analysis (****, *P* < 0.0001).

As shown in Figure 3A, the accuracy of variant calling in the MCRs was much lower than that in flanking regions, and the false discovery rates are much higher in the MCRs than in flanking regions. Increasing either read length or sequencing depth can improve the quality of variant calling, but the accuracy of variant calling in MCRs remains comparably lower, and the false discovery rates in MCRs remain much higher (Figure 3A and Table S8). Even with 150-bp paired-end sequencing (PE150) and 100× depth, the variant identification accuracy is only 35 percent, and the false negative rate remains as high as 60 percent.

A high false discovery rate and low accuracy for the variants in MCRs seems unavoidable with current HTS approaches. Because the minimum length of our MCRs (≥ 300 bp) is longer than the read length commonly used in HTS, reads originating from one MCS can be easily mapped to either locus. Consequently, a genetic variant carried in the original MCR could be mis-identified as the other and cause a false positive result. The mis-alignment could also decrease the allele frequency for the true variant position, thus would cause a false negative result.

To assess whether different combinations of read lengths and sequencing depths affect the accuracy, we performed a *t*-test for 160 simulations (Figure 3B). In general, changes in variant calling accuracy are significant between different sequencing depths and/or different read lengths. However, although increasing sequencing depth from 50× to 100× significantly increases the variant calling accuracy, the accuracies of both strategies are similar to PE150 and 10× depth. These all-to-all pairwise comparisons among different sequencing strategies may be helpful for the community.

The low accuracy and high false discovery rate for variant identification in the MCRs using HTS methods poses a serious challenge for related genetic and genomic studies. Our results also suggest that genetic variants in the MCRs need to be further validated using approaches that offer longer sequencing reads. Furthermore, extra caution is needed for clinical diagnosis of the disease-causing mutations in these regions. To facilitate research in this field, we have provided a Python script, which takes a VCF format input file and outputs the variants in the MCRs (File S2).

## Discussion

The complete sequence of the human genome was believed to represent the dawn of decoding genetic diseases [44], [45]. However, with one-thousandth mutational rate for each individual, precisely identifying disease-causing variants remains a major challenge [46], [47]. MCRs intrinsically make variant identification even more difficult, complicating the task of unambiguously assigning variants to right genomic loci. Our findings further suggest that MCRs participate in protein-coding genes, DSBs, and long-range chromatin interactions, raising the possibility that variants within MCRs may play critical roles in a cell. Indeed, the identification of thousands of pathogenic variants within the MCRs in the ClinVar database validates the importance of genetic alterations in the MCRs in human health. We hope that longer sequencing reads and higher sequencing fidelity will help researchers identify more important mutations in these long identical regions in the future. Accurately identifying the genetic variants in the MCRs may help us unravel the molecular mechanisms of many more Mendelian traits and diseases.

Based on a wealth of knowledge of disease-causing variants, HTS has been widely used in clinical settings for diagnostic purposes [48], [49]. Our result suggests that HTS methods may lead to mis-diagnosis when the genetic alterations fall within MCRs. For now, Sanger sequencing may be a better and safer solution for these variants [50].

Finally, our results suggest that the records of genetic variants from healthy individuals, such as the volunteers of the 1000 Genomes project and the control groups in GWAS studies, may need to be reinvestigated. Further validation is needed for the sequence variations, especially for those acquired from short reads and/or low sequencing depths.

## Conclusion

In this study, we identify a set of MCSs, each of which corresponds to at least two MCRs in distinct loci of the human genome. These MCRs account for roughly 1.98% of the genome and may exert important biological functions through the genes with which they overlap or by affecting long-range chromatin interactions. Our data suggest that further exploration of these regions may have a great impact on biological and clinical research. However, the nature of these duplicated regions may impede the success of genomic studies based on the commonly used HTS technology. The data we present here may serve as a warning that, in order to avoid mis-interpretation caused by the false discovery of genetic variants, extra measures and cautions must be taken in the future.

## Materials and methods

### Identification of MCSs

These tiling sequences with a length of 300 bp and a 1 bp interval were generated for each chromosome and the mitochondrial genome and were mapped back to the same human genome via Burroughs-Wheeler Aligner. Sequences that mapped exactly to multiple loci were extracted as “seeds” for MCSs. If consecutive seeds were perfectly mapped to different loci in succession, they were merged until the continuity was interrupted. The resultant sequences were thus referred to as MCSs. The corresponding regions that MCSs resided were deemed as MCRs.

### GO analysis

The R packages of clusterProfiler (version 3.8.1) (http://bioconductor.org/packages/release/bioc/html/clusterProfiler.html) and org.Hs.eg.db (version 3.6.0) (http://bioconductor.org/packages/release/data/annotation/html/org.Hs.eg.db.html) in Bioconductor were downloaded and installed. GO analyses were performed by inputting the Ensemble IDs of MCR-overlapping genes. The threshold for the enriched terms was set as an adjusted *P* value of less than 0.05.

### Enrichment test

We randomly chose a set of non-overlapping chromosome regions with the same number and length distribution as the MCRs. Mutations from ClinVar, GWAS, and dbSNP falling in these simulated regions were collected. The number of simulated regions in the genome related to chromatin interaction and DSBs were counted. We performed a Chi-Squared test compared to the results from the actual MCRs and computed a *P* value. In all, 1000 independent simulations were executed. A two-tailed *P* value <0.001 was considered statistically significant.

### Extracting variants records from public databases

Genetic variant datasets were downloaded from ClinVar (ftp://ftp.ncbi.nlm.nih.gov/pub/clinvar/tab_delimited/variant_summary.txt.gz), the 1000 Genomes Project phase 3 (ftp://ftp.1000genomes.ebi.ac.uk/vol1/ftp/release/20130502/), TCGA (dated Jan 28, 2016, http://firebrowse.org/), and the NHGRI-EBI catalog of genome-wide association studies (https://www.ebi.ac.uk/gwas/docs/file-downloads). The genomic coordinates of interspersed repeats, segmental duplication, long-range chromatin interactions, and DSBs were downloaded from their respective websites (http://www.repeatmasker.org/, http://humanparalogy.gs.washington.edu/, https://www.encodeproject.org/matrix/?type=Experiment&assay_title=ChIA-PET, and http://genome.ucsc.edu/ENCODE/dataMatrix/encodeDataMatrixHuman.html). The coordinates from different genome versions were converted using the Batch Coordinate Conversion (LiftOver) tool from the UCSC Genome Browser website (genome.ucsc.edu). Genetic variants in the MCRs were extracted using a set of in-house Python scripts based on the unified coordinates.

### Simulation of HTS datasets

We randomly introduced SNVs in the MCRs and their flanking regions of the same length at a rate of one SNV per kb. The genotype of every simulated SNV was recorded. Using wgsim, we simulated 75-bp and 150-bp paired-end (PE75 and PE150, respectively) HTS data with sequencing depths of 10×, 30×, 50×, and 100×. To ensure the accuracy and reliability of the results, 20 independent simulations with different read lengths and sequencing depths were performed. Variants were called with the same pipeline and fixed parameters.

### Variant calling procedure

Low-quality simulated reads were removed based on a unified criterion for each dataset. The clean reads were then mapped to the human genome via Burrows-Wheeler Aligner 0.7.10. Only uniquely mapped reads were retained for subsequent analyses. Variants were detected using the Genome Analysis Toolkit (version 3.4-46-gbc02625). To avoid the possible bias in the variant calling procedure, we used default parameters for all simulated datasets.

### Calculations of variant accuracy, false positive, and false negative values

Taking advantage of known variants in the simulated data, we deemed the imputed variants by wgsim as “true variants” and the variants identified by the aforementioned procedure as “called variants.” The “called variants” that were not identified among the “true variants” were defined as false positive variants. The variants in the “true variants” that were not identified among the “called variants” were defined as false negative variants. The intersection of the “true variants” and the “called variants” were defined as true positive variants. The accuracy of each simulated dataset was calculated as the percent of true positive variants in the “called variants.” The false positive rate was calculated as the percent of false positive variants in the “called variants.” The false negative rate was calculated as the percent of false negative variants in the “true variants.” The statistical significance values among simulations were calculated using independent samples *t*-test in R version (version 3.4.2).

## CRediT author statement

**Jing Sun:** Investigation, Formal analysis, Software, Visualization, Writing - original draft. **Yanfang Zhang:** Investigation, Formal analysis, Software, Visualization, Writing - original draft. **Minhui Wang:** Investigation. **Qian Guan:** Investigation. **Xiujia Yang:** Investigation, Formal analysis, Software, Visualization, Writing - original draft. **Jin Xia Ou:** Investigation. **Mingchen Yan:** Investigation, Formal analysis, Software, Visualization, Writing - original draft. **Chengrui Wang:** Investigation, Formal analysis, Software, Visualization, Writing - original draft. **Yan Zhang:** Investigation, Formal analysis, Software, Visualization, Writing - original draft. **Zhi-Hao Li:** Formal analysis. **Chunhong Lan:** Project administration. **Chen Mao:** Formal analysis. **Hong-Wei Zhou:** Writing - review & editing. **Bingtao Hao:** Writing - review & editing. **Zhenhai Zhang:** Conceptualization, Supervision, Project administration, Funding acquisition. All authors read and approved the final manuscript.

## Competing interests

The authors declare that they have no competing interests.

## Supplementary material

Supplementary File 1 **The sequences of the MCSs identified in this****study**

Supplementary File 2 Python script for extracting variants that fall in the MCRs

Supplementary Figure S1 **The distribution of MCRs and RepeatMasker regions on the genome A.** Distribution of the repeat elements in the regions defined as MCRs and RepeatMasker region. **B.** Distribution of the MCRs and RepeatMasker regions on the genome. LINE, long interspersed nuclear element; SINE, short interspersed nuclear element; LTR, long terminal repeat; L1, LINE-1; L2, LINE-2; ERV1, endogenous retrovirus 1; ERVL, ERV-related; MaLR, mammalian apparent LTR retrotransposon; MIR, mammalian-wide interspersed repeat.

Supplementary Figure S2 **The MCR lengths and distributions on each chromosome A.** Relationship between the length of a chromosome (on the X axis) and the total length of MCRs contained on the respective chromosome (Y axis). **B.** Distribution of intra-chromosomal and inter-chromosomal MCRs on each chromosome.

Supplementary Figure S3 **GO analysis of MCR-overlapping genes** GO terms enrichment in the MCR-overlapping genes was analyzed (adjusted *P* value <0.05). **A.** Biological process. **B.** Cellular component. **C.** Molecular function. Number at the end of each bar means the number of genes that are enriched in such term.

Supplementary Figure S4 **Variant frequency per kb in the public database A.** SNV frequency in the 1000 Genomes Project database. **B.** Mutation frequency in the ClinVar database. **C.** Somatic mutation frequency in The Cancer Genome Atlas. SNV, single-nucleotide variant.
