# Supplementary material for: The Biological Significance of Multi-copy Regions and Their Impact on Variant Discovery
Source: Genomics Proteomics Bioinformatics. 2020 Aug 19;18(5):516–24. doi: 10.1016/j.gpb.2019.05.004 (PMC8377240; doi:10.1016/j.gpb.2019.05.004)
Supplement: Supplementary Figure S1 — The distribution of MCRs and RepeatMasker regions on the genome A. Distribution of the repeat elements in the regions defined as MCRs and RepeatMasker region. B. Distribution of the MCRs and RepeatMasker regions on the genome. LINE, long interspersed nuclear element; SINE, short interspersed nuclear element; LTR, long terminal repeat; L1, LINE-1; L2, LINE-2; ERV1, endogenous retrovirus 1; ERVL, ERV-related; MaLR, mammalian apparent LTR retrotransposon; MIR, mammalian-wide interspersed repeat. [file mmc3.pptx]

## Slide 1
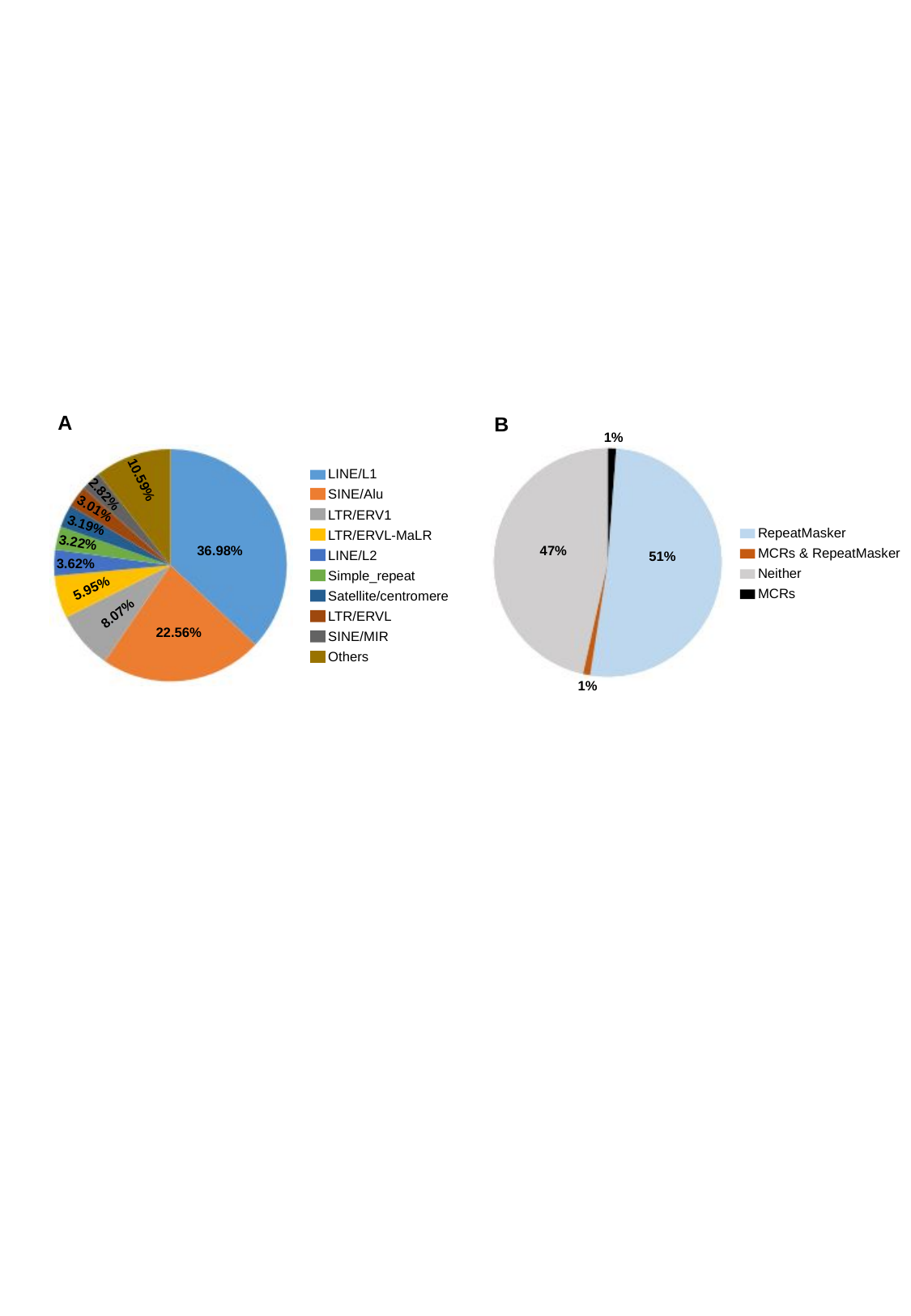

A
B
1%
RepeatMasker
MCRs & RepeatMasker
Neither
MCRs
 47%
51%
1%
LINE/L1
SINE/Alu
LTR/ERV1
LTR/ERVL-MaLR
LINE/L2
Simple_repeat
Satellite/centromere
LTR/ERVL
SINE/MIR
Others
10.59%
2.82%
3.01%
3.19%
3.22%
36.98%
3.62%
5.95%
8.07%
 22.56%
