# Supplementary material for: The Biological Significance of Multi-copy Regions and Their Impact on Variant Discovery
Source: Genomics Proteomics Bioinformatics. 2020 Aug 19;18(5):516–24. doi: 10.1016/j.gpb.2019.05.004 (PMC8377240; doi:10.1016/j.gpb.2019.05.004)
Supplement: Supplementary Figure S2 — The MCR lengths and distributions on each chromosome A. Relationship between the length of a chromosome (on the X axis) and the total length of MCRs contained on the respective chromosome (Y axis). B. Distribution of intra-chromosomal and inter-chromosomal MCRs on each chromosome. [file mmc4.pptx]

## Slide 1
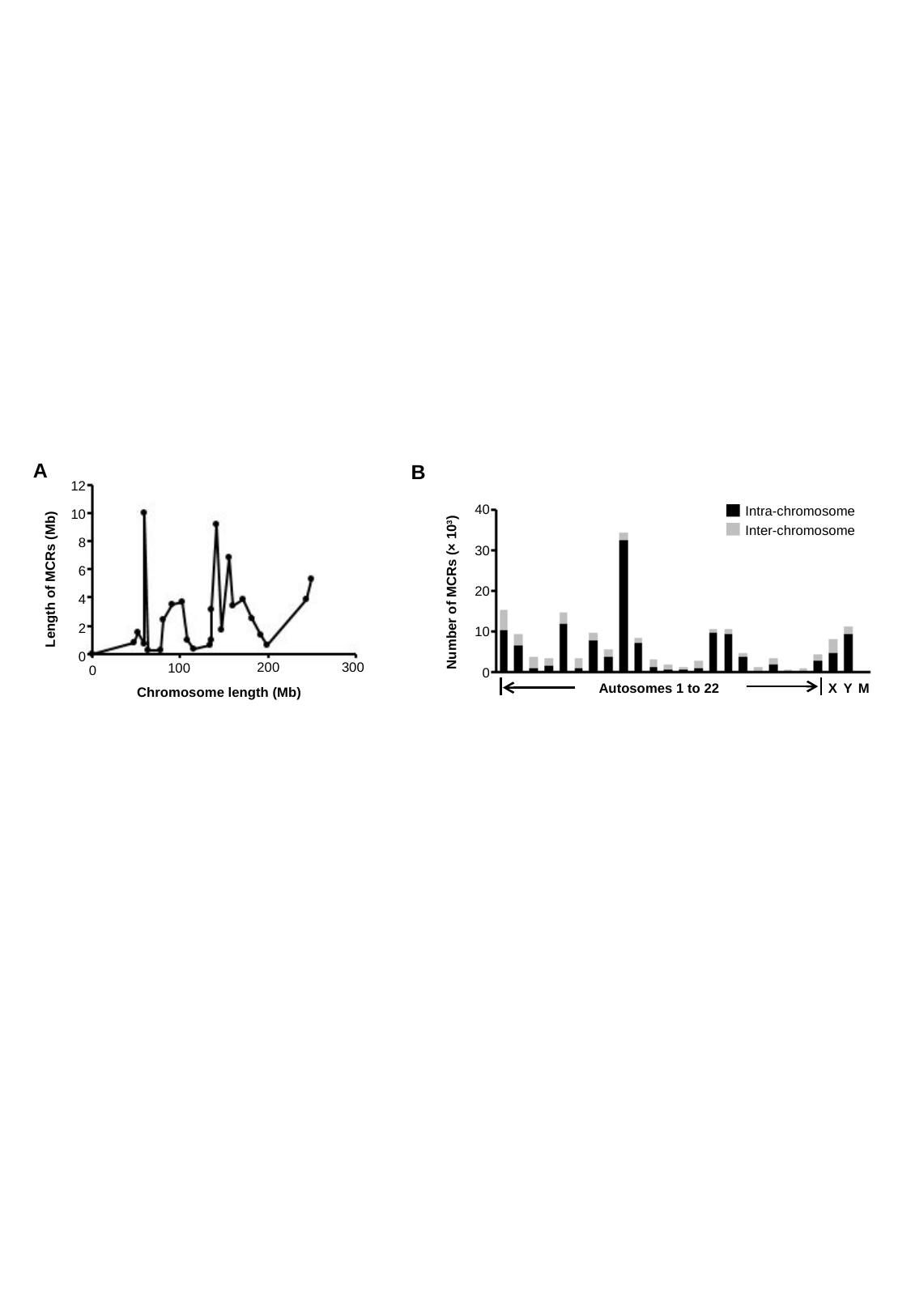

A
B
40
Intra-chromosome
Inter-chromosome
30
20
Number of MCRs (× 103)
10
0
X
Y
M
Autosomes 1 to 22
12
10
8
6
Length of MCRs (Mb)
4
2
0
200
300
100
0
Chromosome length (Mb)
