# Supplementary material for: The Biological Significance of Multi-copy Regions and Their Impact on Variant Discovery
Source: Genomics Proteomics Bioinformatics. 2020 Aug 19;18(5):516–24. doi: 10.1016/j.gpb.2019.05.004 (PMC8377240; doi:10.1016/j.gpb.2019.05.004)
Supplement: Supplementary Figure S3 — GO analysis of MCR-overlapping genes GO terms enrichment in the MCR-overlapping genes was analyzed (adjusted P value <0.05). A. Biological process. B. Cellular component. C. Molecular function. Number at the end of each bar means the number of genes that are enriched in such term. [file mmc5.pptx]

## Slide 1
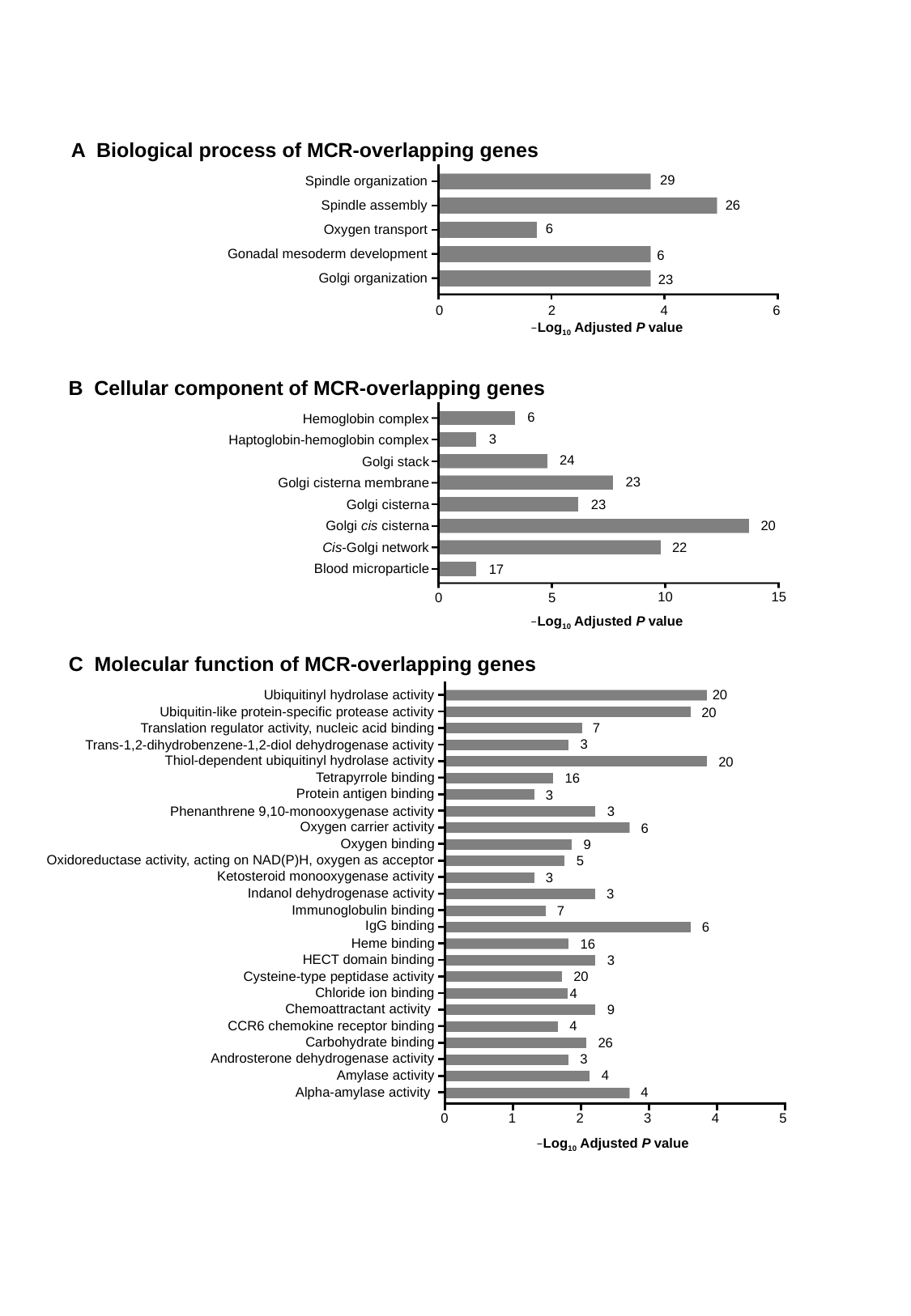

A Biological process of MCR-overlapping genes
29
Spindle organization
Spindle assembly
26
6
Oxygen transport
Gonadal mesoderm development
6
Golgi organization
23
0
2
4
6
–Log10 Adjusted P value
B Cellular component of MCR-overlapping genes
6
Hemoglobin complex
3
Haptoglobin-hemoglobin complex
24
Golgi stack
23
Golgi cisterna membrane
Golgi cisterna
23
Golgi cis cisterna
20
Cis-Golgi network
22
Blood microparticle
17
10
15
0
5
–Log10 Adjusted P value
C Molecular function of MCR-overlapping genes
Ubiquitinyl hydrolase activity
20
Ubiquitin-like protein-specific protease activity
20
Translation regulator activity, nucleic acid binding
7
3
Trans-1,2-dihydrobenzene-1,2-diol dehydrogenase activity
Thiol-dependent ubiquitinyl hydrolase activity
20
Tetrapyrrole binding
16
Protein antigen binding
3
3
Phenanthrene 9,10-monooxygenase activity
Oxygen carrier activity
6
Oxygen binding
9
Oxidoreductase activity, acting on NAD(P)H, oxygen as acceptor
5
Ketosteroid monooxygenase activity
3
Indanol dehydrogenase activity
3
Immunoglobulin binding
7
IgG binding
6
Heme binding
16
HECT domain binding
3
Cysteine-type peptidase activity
20
Chloride ion binding
4
Chemoattractant activity
9
CCR6 chemokine receptor binding
4
Carbohydrate binding
26
Androsterone dehydrogenase activity
3
Amylase activity
4
4
Alpha-amylase activity
0
1
2
3
4
5
–Log10 Adjusted P value
