# Supplementary material for: The Biological Significance of Multi-copy Regions and Their Impact on Variant Discovery
Source: Genomics Proteomics Bioinformatics. 2020 Aug 19;18(5):516–24. doi: 10.1016/j.gpb.2019.05.004 (PMC8377240; doi:10.1016/j.gpb.2019.05.004)
Supplement: Supplementary Figure S4 — Variant frequency per kb in the public database A. SNV frequency in the 1000 Genomes Project database. B. Mutation frequency in the ClinVar database. C. Somatic mutation frequency in The Cancer Genome Atlas. SNV, single-nucleotide variant. [file mmc6.pptx]

## Slide 1
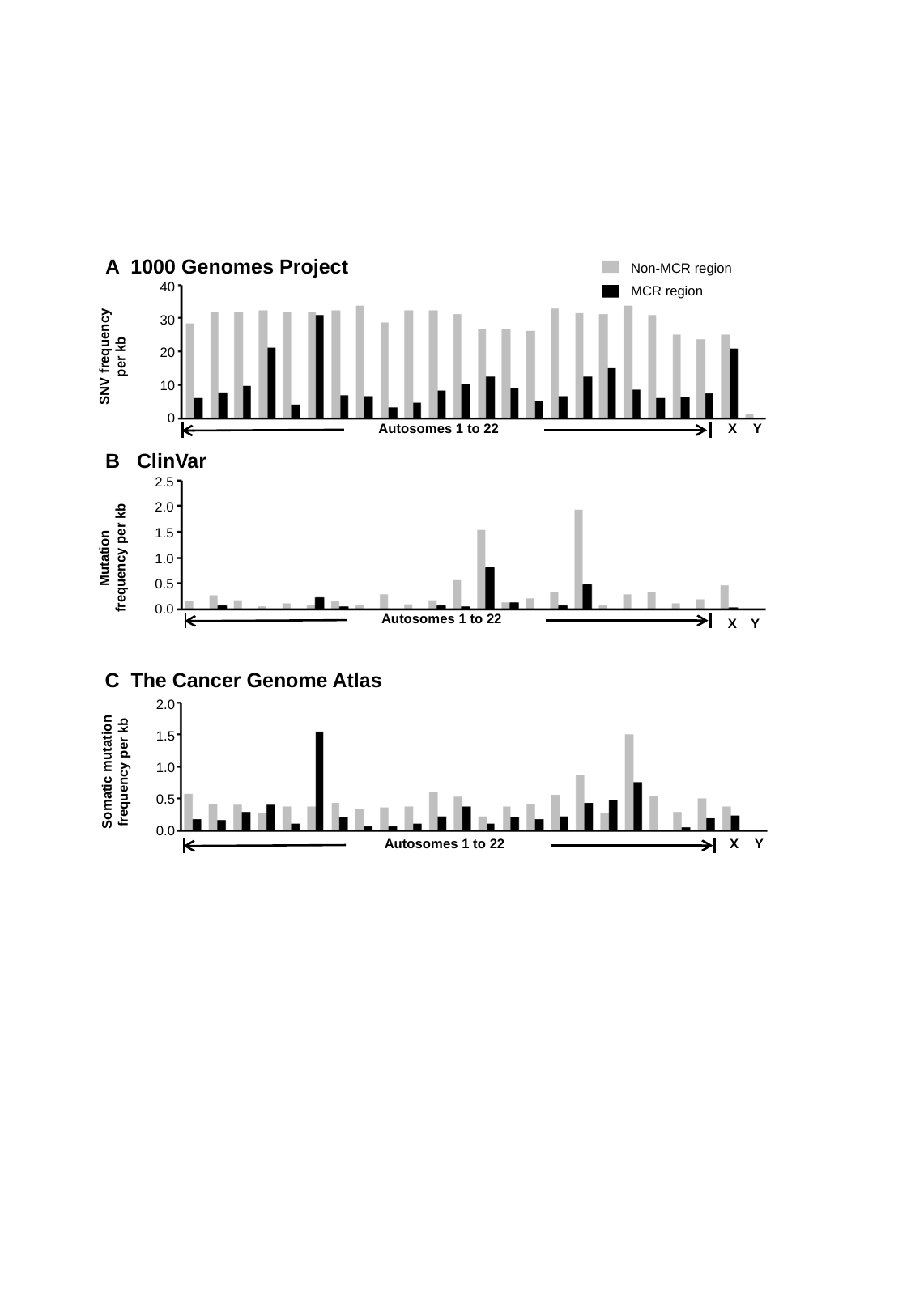

A 1000 Genomes Project
Non-MCR region
MCR region
40
SNV frequency
per kb
30
20
10
0
Autosomes 1 to 22
X
Y
B ClinVar
2.5
Mutation frequency per kb
2.0
1.5
1.0
0.5
0.0
Autosomes 1 to 22
X
Y
C The Cancer Genome Atlas
Somatic mutation frequency per kb
2.0
1.5
1.0
0.5
0.0
Autosomes 1 to 22
X
Y
